# New Data, Old Story: Molecular Data Illuminate the Tribal Relationships among Rove Beetles of the Subfamily Staphylininae (Coleoptera: Staphylinidae)

**DOI:** 10.3390/insects11030164

**Published:** 2020-03-04

**Authors:** Erik Tihelka, Margaret K. Thayer, Alfred F. Newton, Chenyang Cai

**Affiliations:** 1 Department of Animal Science, Hartpury College, Hartpury GL19 3BE, UK; erik.tihelka@hartpury.ac.uk; 2 Negaunee Integrative Research Center, Field Museum of Natural History, Chicago, IL 60605, USA; mthayer@fieldmuseum.org (M.K.T.); anewton@fieldmuseum.org (A.F.N.); 3 State Key Laboratory of Palaeobiology and Stratigraphy, Nanjing Institute of Geology and Palaeontology, and Center for Excellence in Life and Paleoenvironment, Chinese Academy of Sciences, Nanjing 210008, China; 4 School of Earth Sciences, University of Bristol, Life Sciences Building, Tyndall Avenue, Bristol BS8 1TQ, UK

**Keywords:** Staphylininae, phylogeny, classification, Staphylinini, Xantholinini, *Arrowinus*, *Coomania*, Platyprosopus

## Abstract

The megadiverse subfamily Staphylininae traditionally belonged to the best-defined rove beetle taxa, but the advent of molecular phylogenetics in the last decade has brought turbulent changes to the group’s classification. Here, we reevaluate the internal relationships among the tribes of Staphylininae by implementing tree inference methods that suppress common sources of systematic error. In congruence with morphological data, and in contrast to some previous phylogenetic studies, we unambiguously recover Staphylininae and Paederinae as monophyletic in the traditional sense. We show that the recently proposed subfamily Platyprosopinae (*Arrowinus* and *Platyprosopus*) is a phylogenetic artefact and reinstate *Arrowinus* as a member of Arrowinini stat. res. and *Platyprosopus* as a member of Platyprosopini stat. res. We show that several recent changes to the internal classification of the subfamily are phylogenetically unjustified and systematically unnecessary. We, therefore, reestablish Platyprosopini, Staphylinini, and Xantholinini as tribes within Staphylininae (all stat. res.) and recognize Coomaniini as a tribe (stat. nov.) rather than subfamily. Consequently, the traditional ranks of the subtribes Acylophorina, Afroquediina, Amblyopinina, Antimerina, †Baltognathina, Cyrtoquediina, Erichsoniina, Hyptiomina, Indoquediina, Quediina, and Tanygnathinina are restored (all stat. res.). We review the current classification of Staphylininae and discuss sources of incongruence in multigene phylogenies.

## 1. Introduction

Rove beetles (Staphylinidae) are recognized as the most diverse metazoan family in terms of their astonishing species richness [1]. With over 8700 described species in some 350 genera, the cosmopolitan subfamily Staphylininae represents the third largest rove beetle subfamily and accounts for 14% of its biodiversity [2,3]. The subfamily includes some of the largest and most charismatic rove beetles that have captured the attention of naturalists for over two centuries [4,5]. For much of its history, the subfamily Staphylininae has been relatively well-defined and accepted as monophyletic on the basis of staphylinine pupae being obtect and the larvae possessing a large triangular eusternum on the prothorax, along with other unique characters of immatures and adults [2,3,6,7,8,9]. The monophyly of Staphylininae was also supported in formal morphological and molecular analyses [10,11,12,13]. A sister relationship between Staphylininae and Paederinae is well-supported by multiple adult [14,15] and larval characters [16], along with analyses of six and two genes [3,13].

As is typical of many large and morphologically diverse taxa, the evolutionary relationships among staphylinine tribes remain poorly resolved, with morphological and molecular analyses yielding ambivalent results [3,10,12,13,17,18,19,20,21]. Notably, molecular studies conducted principally over the last four years have recovered several unexpected topologies within Staphylininae, with implications for the classification of this megadiverse subfamily. Two recent studies based on five [19] and six [17] genes and a single morphological phylogenetic analysis of extant and fossil taxa [20] have recovered Paederinae nested within Staphylininae, rendering the latter paraphyletic.

Recently, the relationship between Staphylininae and Paederinae was restudied by Cai et al. [3] and Żyła and Solodovnikov [21]; both studies included six genes for representatives of all staphylinine tribes. Cai et al. [3] demonstrated that the paraphyly of Staphylininae is a phylogenetic artefact resulting from limited taxon sampling within Staphylininae in earlier studies and the selection of misleading outgroup taxa that lacked sequences for informative genes. While Staphylininae is now well-supported as monophyletic in the traditional sense [3,21], taxonomic changes were introduced by Żyła and Solodovnikov [21] that divided Staphylininae into four new subfamilies. Further questions surround the relationship between the enigmatic genera *Arrowinus* and *Platyprosopus*. These two genera were recovered as sisters to each other and were included in a redefined Platyprosopinae [21], despite lacking shared morphological apomorphies [10].

The selection of tree inference models can have a major impact on the outcome of phylogenetic analyses. Different models make different assumptions about the nature of the analyzed molecular data and violations of these assumptions may lead to the recovery of misleading topologies, albeit often with high statistical support. To test for sources of phylogenetic incongruence in past analyses of Staphylininae caused by model selection, we used both site-heterogeneous and site-homogeneous models to analyze our data. Unlike the latter, site-heterogeneous models account for compositional heterogeneity across sites, providing a higher degree of realism. We show that the unexpected results in past analyses of Staphylininae can be resolved when more sophisticated tree inference models are used. We discuss our results in relation to the recently proposed changes in the classification of Staphylininae.

## 2. Materials and Methods 

### 2.1. Taxon Sampling and Sequence Alignment

To test the inter-relationships among the tribes of Staphylininae, we used the most comprehensive dataset available for this subfamily to date, including 56 ingroup taxa [21]. All extant tribes of Staphylininae in the traditional sense were represented as follows: Arrowinini (one genus, two species), Coomaniini (one genus, one species), Diochini (one genus, five species), Maorothiini (one genus, two species), Othiini (two genera, five species), Platyprosopini (one genus, two species), Xantholinini (12 genera, 12 species), and Staphylinini (25 genera and 27 species). GenBank sequence numbers are the same as those provided in [21]. We also reanalyzed a second dataset with a more restricted taxon sampling, provided by Cai et al. [3], focusing primarily on Staphylinini. This second dataset consisted of the following 84 ingroup taxa: Arrowinini (one genus, two species), Diochini (one genus, one species), Maorothiini (one genus, one species), Othiini (two genera and three species), Platyprosopini (one genus, one species), Staphylinini (43 genera and 72 species), and Xantholinini (four genera and four species). In both datasets, the nuclear protein-coding genes *arginine kinase* (*ArgK*), *carbamoyl-phosphate synthetase* (*CAD*), *topoisomerase I* (*TP*), and *wingless* (*Wg*); the mitochondrial protein-coding *cytochrome oxidase I* (*COI*); and the nuclear ribosomal *28S* were used.

All sequences were obtained from GenBank using the Batch Entrez tool. Protein-coding genes were aligned with respect to their codon structure in mega x 10.0.5 with the ‘MUSCLE’ tool [22]. The ribosomal 28S gene was aligned using the Q-INS-I algorithm in mafft on xsede 7.402 [23,24] available through the CIPRES Science Gateway [25]. The gene sequences were concatenated in SequenceMatrix 1.8 [26]. The highly heterogeneous third codon of *COI*, which has been suggested to suffer from saturation and is hence regarded as uninformative for inferring deeper phylogenies [27], was excluded from the analyses.

### 2.2. Outgroup Selection

We used two different sets of outgroups in our analyses of the Żyła and Solodovnikov [21] data: (1) the same set of outgroups as in the original study, and (2) a more restricted set of outgroup taxa devised by us. Żyła and Solodovnikov [21] used 25 outgroup taxa: Euaesthetinae (one genus, one species), Megalopsidiinae (one genus, one species), Olisthaerinae (one genus, three species), Oxyporinae (one genus, one species), Paederinae (15 genera and 15 species), Pseudopsinae (one genus, one species), Steninae (one genus, one species), and Tachyporinae (two genera, two species). Considering that the focus of our present analyses is the inter-relationships within Staphylininae, such a wide sampling of outgroup taxa does not appear necessary. In fact, the inclusion of distantly related outgroups can lead to long branch attraction [28,29]. Moreover, incomplete gene sampling for the genera *Pseudopsis* and *Olisthaerus* could obscure character polarity and lead to misleading topologies [3]. We therefore decided to limit the outgroup selection to the more closely related Paederinae along with *Lordithon lunulatus* as a representative of Tachyporinae and *Oxyporus femoralis* as a representative of Oxyporinae. The more distantly related subfamilies Euaesthetinae, Megalopsidiinae, Olisthaerinae, Pseudopsinae, and Steninae were excluded. 

For the Cai et al. [3] data, we used the original outgroup consisting of six paederines and *Oxyporus femoralis* (Oxyporinae).

### 2.3. Model Selection, Data Partitioning and Phylogenetic Analyses

The site-heterogeneous model CAT-GTR was implemented in phylobayes mpi 1.7 [30,31] run on the University of Bristol BlueCrystal Phase3 Cluster. Two independent Markov chain Monte Carlo (MCMC) chains were run until convergence. The bpcomp program was used to generate the largest (maxdiff) and mean (meandiff) values. The chains were considered to have converged when maxdiff was <0.3. Both the Żyła and Solodovnikov [21] data and the Cai et al. [3] data were reanalyzed using the CAT-GTR model.

For analyses with site-homogeneous models, both maximum likelihood (ML) and Bayesian inference (BI) approaches were used as implemented in iq-tree and MrBayes, respectively. For analysis by site-homogeneous models, the dataset was partitioned by gene and by codon position for the protein-coding genes. Models were selected using PartitionFinder 2.1.1 [32]. The ‘greedy’ algorithm was used to search ‘all’ models under the Bayesian information criterion, with branch lengths unlinked. This resulted in the following partitioning scheme: (1) *28S*: GTR+I+G; (2) position 1 of *ArgK*: TVMEF+I+G; (3) position 2 of *ArgK*: TVMEF+I+G; (4) position 3 of *ArgK*: GTR+I+G; (5): position 1 of *CAD*: SYM+G; (6) position 2 of *CAD*, position 2 of *Wg*, position 2 of *TP*: GTR+I+G; (7) position 3 of *CAD*: TRN+I+G; (8) position 1 of *COI*: GTR+I+G; (9) position 2 of *COI*: TVM+I+G; (10) position 1 of *TP*, position 1 of *Wg*: SYM+I+G; (11) position 3 of *Wg*: K80+G; (12) position 3 of *Wg*: GTR+I+G. ML analyses were performed using iq-tree v.1.6 [33]. Each run in MrBayes v.3.2.6 x86 [34] consisted of one cold and three heated chains, run for six million generations or until convergence. Node support was assessed with 1000 bootstrap pseudoreplicates. Only the Żyła and Solodovnikov [21] data were reanalyzed with site-homogeneous models, as the Cai et al. [3] data were already analyzed using methods identical to those used by us.

Clades with Bayesian posterior probability (BPP) ≥ 0.95 or ML bootstrap (BS) values > 80 were considered as strongly supported; clades with BPP of 0.90–0.94 or BS of 70–80 were considered as moderately supported; clades with BPP of 0.85–0.89 or BS of 50–69 were considered to be weakly supported; and clades with BPP < 0.85 or BS < 50 were considered to be unsupported [3].

## 3. Results

### 3.1. Monophyly of Staphylininae and Paederinae

**Figure 1:**
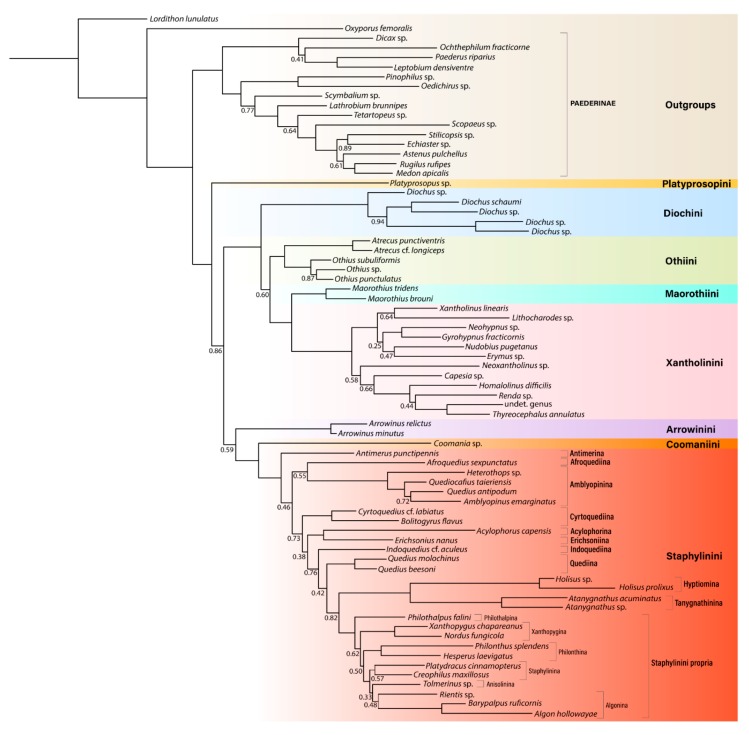
Intertribal relationships within the subfamily Staphylininae based on data from Żyła and Solodovnikov [21] reanalyzed under a site-heterogeneous CAT-GTR model with distantly related outgroups excluded. Unlabeled nodes are strongly supported (BPP ≥ 0.95). Posterior probabilities smaller than 0.95 are reported below each node.

The phylogeny of Staphylininae was estimated using a site-heterogeneous model (Figure 1), and site-homogeneous ML and BI models [3,21]. The site-heterogeneous model yielded well-resolved topologies with high statistical support at deep nodes. Regardless of the inference model used or the dataset analyzed, Staphylininae was always well-supported as monophyletic, with Paederinae forming a sister group. This result is congruent with classical morphology-based classification schemes [2,7,9].

The apparent paraphyly of Staphylininae found in previous molecular studies [17,19] was caused in part by limited taxon sampling and because a key outgroup taxon used in the analyses, *Pseudopsis*, lacked data for the *Wg* gene which is important for recovering relationships above the subtribe level [3]. Upon correcting these ambiguities, Staphylininae has been consistently recovered as monophyletic [3,21]. The apparent paraphyly of Staphylininae reported by a single morphological phylogeny [20] was, in fact, statistically unsupported. As such, we interpret Staphylininae and Paederinae as uncontroversially monophyletic.

### 3.2. Phylogeny of Staphylininae

#### 3.2.1. Position of Arrowinini

**Figure 2:**
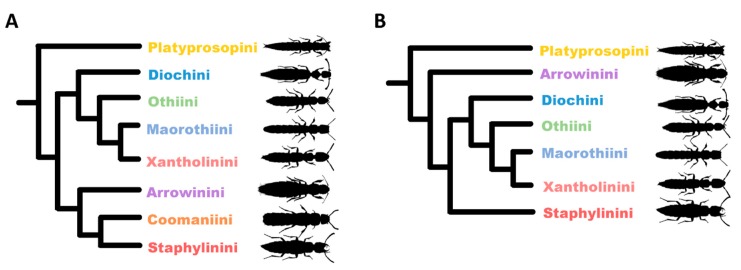
Summary of competing hypotheses on the intertribal relationships within Staphylininae showing the uncertain position of the tribe Arrowinini. (**A**) Topology recovered by reanalyzing data from Żyła and Solodovnikov [21] with a site-heterogeneous CAT-GTR model (Figure 1). (**B**) Topology recovered by reanalyzing data from Cai et al. [3] with a site-heterogeneous CAT-GTR model [21] with a site-heterogeneous CAT-GTR model. Note that Coomaniini was not sampled in the latter dataset.

When analyzed with the site-heterogeneous model, all staphylinine tribes in the traditional sense [3] were recovered as monophyletic with high statistical support. The analyses resulted in two competing hypotheses of Staphylininae relationships, varying with respect to the position of the tribe Arrowinini (Figure 2). The topology where Arrowinini constitutes the sister group to Coomaniini and Staphylinini—Platyprosopini (((Diochini (Othiini (Maorothiini, Xantholinini))), (Arrowinini (Coomaniini, Staphylinini))))—was recovered by the phylobayes analyses of the Żyła and Solodovnikov [21] data, regardless of the outgroup used (although support was higher when distant outgroups were included). On the other hand, a more basal position of Arrowinini, as a sister to the rest of Staphylininae except Platyprosopini, was supported by the phylobayes reanalysis of the Cai et al. [3] and the Żyła and Solodovnikov [21] data. The results were the same when the data were analyzed under site-homogeneous models; the Żyła and Solodovnikov [21] dataset favored a derived position of Arrowinini, while the previously published site-homogeneous analyses of Cai et al. [3] data recovered a more basal position of the tribe.

Indeed, past opinions on the position of the relictual and monogeneric South African tribe Arrowinini have been contradictory. While it was originally described as an isolated member of Quediini [35], it was later recognised as a separate lineage and elevated to tribal rank [10]. In analyses of adult morphological characters, Arrowinini was consistently recovered as a sister group to Staphylinini based on a single apomorphic character—the shared presence of prototergal glands [10,20,36]. However, a parsimony analysis of larval characters recovered *Arrowinus* as a sister group to Platyprosopini based on the shared presence of multiple tarsungular spines [10], although the spines in the two genera differed in details and the authors mentioned the possibility of this not being a synapomorphy. Molecular studies have yielded similarly variable results with *Arrowinus* either as the basalmost staphylinine clade together with Platyprosopini [3,17,19,21], sister group to Diochini [12], or close to Staphylinini [13,18]. As the position of the tribe Arrowinini is clearly unstable and contingent upon taxon sampling, we propose that the resolution of this phylogenetic question will require sequencing additional genes to recover more phylogenetic signal.

#### 3.2.2. Are *Arrowinus* + *Platyprosopus* Monophyletic?

Notwithstanding the unclear systematic position of Arrowinini, the relationship between *Arrowinus* and the widely distributed *Platyprosopus*, native to the tropical or warm parts of the Neotropical, Nearctic, Palaearctic, Ethiopian, Madagascan, and Oriental regions [37], is of a particular interest. A sister relationship between these two genera was first proposed by an analysis of larval characters, and adult and larval characters combined, with moderate to weak support [10] and recently recovered again in an analysis of six genes [21]. In the latter analysis, the sister relationship between Arrowinini and Platyprosopini was supported in BI analyses but not in ML analyses. Nevertheless, the authors proposed that Arrowinini and Platyprosopini form a clade, which they elevated to subfamily rank and called ‘Platyprosopinae’. The larval characters thought to unite Arrowinini and Platyprosopini are, in fact, most probably not synapomorphies. Although both taxa share having more than four spines on the tarsunguli, their shape, arrangement, and number differ markedly, indicating that they may have originated independently. Although both genera share seven teeth on the apical margin of the nasale, this character state is not unique and is also found in Amblyopinina. The shared presence of randomly distributed microspines on abdominal terga II–VIII is also not a unique feature and is found in Pseudopsinae [10]. Our site-heterogeneous analyses never recovered *Arrowinus* and *Platyprosopus* as forming a clade, regardless of the dataset analyzed. A clade comprising *Arrowinus* and *Platyprosopus* was, however, recovered when the Żyła and Solodovnikov [21] data were reanalyzed under the simpler site-homogeneous models.

#### 3.2.3. Systematic Position of *Coomania*

*Coomania* is a monotypic genus known from southeast Asia and Oceania [21]. *Coomania tonkinensis* has seldom been seen since the type series was collected in Vietnam in the 1930s [38]. De Rougemont [39] recently rediscovered the genus in Laos and Malaysia, it was mentioned in the Philippines and Australia [21], and we are aware of several additional specimens in the collections of the Field Museum in Chicago and elsewhere which are now under study by Dagmara Żyła. The morphology of *Coomania* is peculiar. While it was previously placed in Diochini, it differs markedly from *Diochus* in the broad and strongly trapezoidal head (though with an exceptionally narrow neck, as in *Diochus*), broad pronotum, and a generally parallel-sided body. It further differs from all Staphylininae in having a cylindrical abdomen without paratergites [39]. Based on its distinctiveness, the genus was recently placed in its own subfamily by Żyła and Solodovnikov [21]. This separation is further reinforced by our results; *Coomania* was strongly supported as a separate group in all analyses, not nested within any of the traditionally defined tribes, regardless of outgroup selection or the type of inference model used.

#### 3.2.4. Unresolved Nodes and Remaining Questions in Staphylininae Phylogeny

Aside from the unclear position of Arrowinini, the overall results correspond well to the most recent analyses of Staphylininae relationships based on six genes [3,21]. However, several shallower relationships differed profoundly. Although addressing the sub-tribal relationships among staphylinines was not the primary goal of our analysis, several incongruences in particular have to be pointed out. Firstly, the position of Afroquediina has been variable: it was recovered as either a sister group to Amblyopinina (phylobayes of Żyła and Solodovnikov’s [21] data with distant outgroups excluded, BPP = 0.55; phylobayes of Cai et al. [3] data, BPP = 0.55) or a sister group to Staphylinini minus Antimerina and Amblyopinina (PHYLOBAYES of Żyła and Solodovnikov’s [21] data with distant outgroups included, BPP = 0.32); all analyses using site-homogeneous models lent support to the sister relationship between Afroquediina and Amblyopinina. The only commonality of these two competing hypotheses is their poor statistical support. Indeed, the position of Afroquediina has seen low statistical support in previous staphylinine phylogenies as well [3,21,40], identifying *Afroquedius* as a notorious rogue taxon. The relationships within Staphylinini sensu Żyła and Solodovnikov [21] (=Staphylinini propria of prior studies) and Xantholinini, and the positions of Indoquediina and Quediina were likewise poorly supported. This situation is likely to change with improved gene sampling.

## 4. Discussion

### 4.1. Sources of Phylogenetic Incongruence

We present a multigene phylogeny of Staphylininae suprageneric relationships. Because the positions of tribes within this traditionally well-defined group have been variable in past studies [3,10,12,13,17,18,19,20,21], we implemented different data selection and analysis approaches to understand possible sources for these inconsistencies. While past studies have relied on site-homogeneous models to analyze molecular data, we have chosen to use a more complex site-heterogeneous model, CAT-GTR, which accounts for compositional heterogeneity across sites. With this feature, CAT-GTR has been shown to fit many molecular datasets better than conventional site-homogeneous models [41]. Because it alleviates common artefacts such as long branch attraction [30,31], site-heterogeneous models have been used to resolve long-standing phylogenetic controversies such as the relationships between prokaryotes, archaea, and eukaryotes [32], or the basal branching order within Metazoa [33,34].

We found that, regardless of the type of inference model selected, the relationships between major tribes and the monophyly of Staphylininae were well-supported. However, the monophyly of a lineage comprising Arrowinini and Platyprosopini was only recovered by simpler site-homogeneous models, while the site-heterogeneous model showed that the two tribes represent a non-monophyletic grade, congruent with morphological evidence. It is possible that the attraction between *Arrowinus* and *Platyprosopus* in site-homogeneous analyses was caused by unbalanced taxon sampling. While the monogeneric Arrowinini and Platyprosopini were represented by only three species in our analyses, most related tribes were represented by more taxa. When only a few taxa of a particular group are included in phylogenies, these taxa are often recovered as long branches or are grouped together with unlikely relatives [42,43]. Site-heterogeneous models have been shown to be able to overcome these types of systematic errors [44,45,46], making them suitable for studying the phylogenetic position of relictual taxa.

While the use of site-heterogeneous models is becoming increasingly common in phylogenomic-scale studies, their use has been limited in analyses using small numbers of genes. Our results show that site-heterogeneous models can considerably improve the results of analyses of even small datasets by recovering relationships that could not be found with simpler BI and ML models. In this sense, the use of more complex models could, to some extent, compensate for low gene sampling. An obvious disadvantage of using site-heterogeneous models is that they can be extremely computationally demanding, especially when large datasets are analyzed.

### 4.2. Taxonomic Implications

In our reanalysis of Staphylininae relationships, the subfamily was unambiguously recovered as monophyletic. All tribes are monophyletic and, with the exception of Arrowinini, their positions were found to be well supported and consistent among the analyzed datasets. Importantly, we demonstrate that *Arrowinus* and *Platyprosopus* do not form a clade. These findings have implications for Staphylininae taxonomy.

Recently, Żyła and Solodovnikov [21] proposed several major taxonomic changes within Staphylininae, dividing it into four subfamilies (Platyprosopinae, Staphylininae, Xantholininae, and the newly proposed Coomaniinae) and elevating several subtribes to tribal level. The authors argued that these taxonomic changes were justified by the large morphological disparity between tribes and their variable biogeographical distribution. Moreover, they argued that the long evolutionary history of Staphylininae [47] should be reflected by the taxonomy of the family. However, given the now well-established monophyly of Staphylininae in the traditional sense [3], these systematic changes appear phylogenetically unjustified. They are moreover taxonomically unnecessary, as the degree of morphological and biogeographic heterogeneity within Staphylininae tribes is no greater than among other beetle subfamilies and, by itself, it cannot be used as a criterion for the erection of new higher taxa. Likewise, the age of Staphylininae is not particularly outstanding when compared to Gyrinidae [48], Ommatidae [49], and indeed dozens of other beetle families and subfamilies [11,50,51,52]. On a practical level, fragmenting one of the best-established rove beetle subfamilies [7] into four has the potential to cause confusion among practicing entomologists and ecologists. This is particularly the case given that there are still many unsequenced and poorly-studied genera in Staphylinini *s. l.* [5,53,54,55] and yet-undescribed fossils near Diochini and *Coomania* from Burmese and Baltic ambers, whose revision in the upcoming years could bring further instability to the classification of rove beetles if the new classification scheme [21] of Staphylininae were to be followed. 

As a result, we did not follow the taxonomic changes by Żyła and Solodovnikov [21] in the present paper and herein formally propose to demote the newly proposed subfamilies back to tribal rank as Coomaniini stat. nov., Platyprosopini stat. res., Staphylinini stat. res., with Xantholinini stat. res., *Platyprosopus* being placed into the monogeneric tribe Platyprosopini, while *Arrowinus* is placed into its own tribe. Hence, the subfamily Staphylininae sensu res., reinstated by us in the classical sense, comprises the following eight extant and one fossil tribes: Arrowinini, Coomaniini, Diochini, Maorothiini, Othiini, Platyprosopini, Staphylinini, †Thayeralinini, and Xantholinini. Furthermore, we reinstate the subtribes within Staphylinini that were elevated to tribal level by Żyła and Solodovnikov [21]: Acylophorina, Afroquediina, Amblyopinina, Antimerina, †Baltognathina, Cyrtoquediina, Erichsoniina, Hyptiomina, Indoquediina, Quediina, and Tanygnathinina (all stat. res.), and return to the use of the informal term “Staphylinini propria” for the remaining six subtribes of Staphylinini (as in [4], and equivalent to “Staphylinini *sensu stricto*” in [21]). An overview of the current classification of Staphylininae is provided in Table 1.

## 5. Conclusions

Our reanalysis of intertribal relationships within Staphylininae in the traditional sense [3], with methods that allow for the detection of sources of phylogenetic incongruence, resulted in a well-supported tree of the subfamily, congruent with morphological evidence. The subfamily and its constituent tribes were recovered as monophyletic. *Arrowinus* was recovered either as a sister group to Coomaniini and Staphylinini, or as a sister group to Staphylininae except Platyprosopini, depending on the dataset analyzed. We demonstrate that the recently proposed clade comprising Arrowinini and Platyprosopini is a phylogenetic artefact that disappears when the data are analyzed using methods that account for unequal taxon sampling [46]. The strong support for both Arrowinini and Staphylinini (in our sense) below and above *Coomania* on the tree supports the independence of the latter. We consequently reinstate Coomaniini, Platyprosopini, Staphylinini, and Xantholinini as tribes and Acylophorina, Afroquediina, Amblyopinina, Antimerina, †Baltognathina, Cyrtoquediina, Erichsoniina, Hyptiomina, Indoquediina, Quediina, and Tanygnathinina as subtribes (all stat nov.).

## Figures and Tables

**Table 1:** Comparison of the classification schemes of Staphylininae of Żyła and Solodovnikov [21] and the revised scheme proposed herein.

Żyła and Solodovnikov [21]	Present Paper
“former subfamily Staphylininae”	
	
XANTHOLININAE Erichson, 1839 Diochini Casey, 1906 Othiini Thomson, 1859 Maorothiini Assing, 2000 Xantholinini Erichson, 1839 PLATYPROSOPINAE Lynch, 1884 †Thayeralinini Solodovnikov & Yue, 2013 Arrowinini Solodovnikov & Newton, 2005 Platyprosopini Lynch, 1884 COOMANIINAE Żyła & Solodovnikov, 2019 STAPHYLININAE Latreille, 1802 †Baltognathini Brunke, Żyła & Solodovnikov, 2019 Afroquediini Brunke, Żyła & Solodovnikov, 2019 Antimerini Brunke, Żyła & Solodovnikov, 2019 Tanygnathinini Reitter, 1909 Hyptiomini Casey, 1906 Amblyopinini Seevers 1944 Erichsoniini Brunke & Solodovnikov, 2016 Acylophorini Outerelo & Gamarra 1985 Cyrtoquediini Brunke & Solodovnikov 2016 Indoquediini Brunke & Solodovnikov, 2016 Quediini Kraatz, 1857 Staphylinini Latreille, 1802 Algonina Schillhammer & Brunke, 2017 Anisolinina Hayashi, 1993 Philonthina Kirby, 1837 Philothalpina Chatzimanolis & Brunke, 2017 Staphylinina Latreille, 1802 Xanthopygina Sharp, 1884	STAPHYLININAE Latreille, 1802 **stat. res.** †Thayeralinini Solodovnikov & Yue, 2013 Platyprosopini Lynch, 1884 **stat. res.** Diochini Casey, 1906 Othiini Thomson, 1859 Maorothiini Assing, 2000 Xantholinini Erichson, 1839 **stat. res.** Arrowinini Solodovnikov & Newton, 2005 Coomaniini Żyła & Solodovnikov, 2019 **stat. nov.** Staphylinini Latreille, 1802 **stat. res.** †Baltognathina Brunke, Żyła & Solodovnikov, 2019 **stat. res.** Antimerina Brunke, Żyła & Solodovnikov, 2019 **stat. res.** Afroquediina Brunke, Żyła & Solodovnikov, 2019 **stat. res.** Amblyopinina Seevers, 1944 **stat. res.** Cyrtoquediina Brunke & Solodovnikov, 2016 **stat. res.** Acylophorina Outerelo & Gamarra, 1985 **stat. res.** Erichsoniina Brunke & Solodovnikov, 2016 **stat. res.** Indoquediina Brunke & Solodovnikov, 2016 **stat. res.** Quediina Kraatz, 1857 **stat. res.** Hyptiomina Casey, 1906 **stat. res.** Tanygnathinina Reitter, 1909 **stat. res.** Staphylinini propria Algonina Schillhammer & Brunke, 2017 Anisolinina Hayashi, 1993 Philonthina Kirby, 1837 Philothalpina Chatzimanolis & Brunke, 2017 Staphylinina Latreille, 1802 Xanthopygina Sharp, 1884
